# Autism Spectrum Disorder and Schizophrenia Are Better Differentiated by Positive Symptoms Than Negative Symptoms

**DOI:** 10.3389/fpsyt.2020.00548

**Published:** 2020-06-11

**Authors:** Dominic A. Trevisan, Jennifer H. Foss-Feig, Adam J. Naples, Vinod Srihari, Alan Anticevic, James C. McPartland

**Affiliations:** ^1^Child Study Center, Yale University School of Medicine, New Haven, CT, United States; ^2^Department of Psychiatry, Mount Sinai Icahn School of Medicine, New York, NY, United States; ^3^Seaver Autism Center for Research and Treatment Mount Sinai Icahn School of Medicine, New York, NY, United States; ^4^Department of Psychiatry, Yale University School of Medicine, New Haven, CT, United States

**Keywords:** autism, schizophrenia, Autism Diagnostic Observation Schedule, Positive and Negative Syndrome Scale, positive symptoms, negative symptoms, symptom overlap

## Abstract

Autism spectrum disorder (ASD) and schizophrenia (SZ) are heterogenous neurodevelopmental disorders that overlap in symptom presentation. The purpose of this study was to specify overlapping symptom domains and to identify symptoms that can reliably differentiate adults with ASD (n = 53), SZ (n = 39), and typical development (TD; n = 40). All participants regardless of diagnosis were administered gold-standard diagnostic assessments of ASD and SZ characteristics including the Autism Diagnostic Observation Schedule (ADOS-2) and the Positive and Negative Syndrome Scale (PANSS). Sensitivity and specificity of the ADOS were assessed using diagnostic cut-off scores. The degree of symptom overlap on these measures between participant groups was analyzed using Analyses of Variance (ANOVAs), Receiver Operating Characteristic (ROC) Curves, and Analyses of Covariance (ANCOVAs) to control for group differences in IQ and sex distributions. The ADOS reliably discriminated ASD and TD adults, but there was a high rate of “false positives” in SZ patients who did not meet the DSM-5 criteria for ASD. To identify the reasons for low specificity in the SZ sample, we categorized ASD and SZ symptoms into ‘positive’ (presence of atypical behaviors) and ‘negative’ (absence of typical behaviors) symptoms. ASD and SZ groups overlapped on negative symptoms largely related to the absence of typical social and communicative behaviors, whereas disorder-specific positive symptoms differentiated ASD and SZ. For example, those with ASD scored higher on restricted and repetitive behaviors and stereotyped language, whereas those with SZ scored higher on psychotic symptoms such as delusions and hallucinations. These results suggest that, when making a differential diagnosis between ASD and SZ, clinicians may benefit from focusing on the presence or absence of positive ASD and SZ symptoms. Standardized measures to classify ASD symptoms into positive and negative symptoms have not yet been developed but represent a potentially viable clinical tool.

## Introduction

Autism spectrum disorder (ASD) and schizophrenia (SZ) are neurodevelopmental disorders with heterogeneous and sometimes, overlapping symptom presentation (1–5). Such heterogeneity and overlap in these and other disorders motivated the National Institute of Mental Health (NIMH) to propose the Research Domain Criteria (RDoC) initiative in an effort to develop new ways of conceptualizing and clustering symptoms within and across different disorders (6). The goal of the RDoC initiative is not to eliminate clinically useful diagnostic categories in the Diagnostic and Statistical Manual of Mental Disorders (DSM-5) (1) but rather to introduce an interrelated classification system that links validated clinical presentations of psychopathology to underlying pathophysiology (6–8). As the DSM-5 categorizes disorders primarily on symptom presentation, a key objective of RDoC is to work towards a classification system that clusters disorders based on biologically meaningful mechanisms—with the ultimate goal of better targeting optimal treatments (7).

ASD and SZ share a long history of diagnostic confusion (9, 10) cf (11). In the late 1800s, Emil Kraepelin popularized the term ‘dementia praecox’ (today known as schizophrenia) to differentiate progressive neurodegenerative disease associated with irreversible loss of cognitive function, from episodic affective disorders such as ‘manic depression’ (12). Shortly thereafter, Sante De Sanctis extended the field of psychiatry to childhood, classifying ‘dementia praecocissima’ as a childhood condition that included psychotic and autistic symptoms by today’s definitions, such as “strangeness of character,” apathy, depressed mood, hallucinations and catatonia (13). The term ‘autism’ was first introduced by Bleuler (14), not as an independent disorder, but as a symptom of schizophrenia, although Bleuler’s definition of autism, the symptom, shares little resemblance to today’s conceptualization of autism, the syndrome (15). Prior to the release of the DSM-III (16) when ASD was first presented as a distinct clinical diagnosis, children now considered to have ASD were commonly diagnosed with “childhood onset schizophrenia” (17)—a childhood disorder characterized by abnormal perceptions of reality in addition to deficits in social functioning (18). Until recently, a rare and severe autistic disorder known as “childhood disintegrative disorder” (CDD; previously known as Heller’s syndrome) characterized by developmental regression, was frequently associated with what is presumed to be paranoia and psychosis (19). Similarly, psychotic symptoms, such as delusions and auditory hallucinations have been observed in less impaired people with ASD and in what was previously termed “Asperger syndrome” (20–22) Although these autism subtypes (CDD and Asperger syndrome) are now subsumed under “autism spectrum disorder” as of DSM-5 (1), these findings demonstrate that psychotic symptoms can be associated with the full spectrum of autism severity.

Though ASD and SZ are now distinct disorders—today’s ASD remains a childhood-onset disorder whereas frank SZ predominantly emerges during young adulthood—the two disorders still share common genetic risk factors and symptom presentations (6, 14, 23–27). Not surprisingly, these disorders frequently co-occur. Recently, a large-scale meta-analysis aggregating close to 2,000,000 million participants found that individuals with ASD are 3.55 times more likely to have a concurrent diagnosis of SZ than controls (28). Further complicating diagnostic precision, both disorders often co-occur with other conditions—ASD frequently co-occurs with disorders such as ADHD, tic disorders, developmental coordination disorder, intellectual disability, depression, and anxiety (29), and SZ frequently co-occurs with panic disorder, posttraumatic stress disorder, obsessive compulsive disorder, depression, anxiety, and substance abuse disorders (30). As such, divisions among diagnostic categories in the DSM-5 can be complex and sometimes arbitrary, highlighting the need for a better understanding of shared risk factors and more nuanced ways of distinguishing symptom presentation across diagnostic categories in order to advance biologically informed research and practice.

SZ features are typically clustered into positive and negative symptom domains (31–33). Within this framework, positive symptoms refer to the presence of atypical symptoms that are not observed in typical development, such as hallucinations or delusions. Negative symptoms refer to the absence or reduction of characteristics or behaviors that are present in typical development, such as flat facial affect, or paucity of communicative gestures. While this clustering of symptoms has long been used in the SZ literature, it was only recently proposed as a viable framework for clustering ASD symptomology (34). Adopting this framework from the SZ literature offers a new way of conceptualizing ASD symptomology that could better capture heterogeneity and allow for a dimensional approach to studying and treating shared and distinct symptomology across overlapping diagnostic categories.

Symptom overlap between ASD and SZ may be most apparent in the negative symptom domain, broadly reflecting deficits in social communication and social–emotional reciprocity. For example, negative symptoms in SZ include flat or blunted affect (*e.g.*, reduced affective sharing, eye contact, facial expressions, and use of gesture), alogia (impoverished speech, perhaps reflecting difficulties with thinking and cognition), avolition/apathy (lack of energy, drive, and interest), anhedonia (lack of social and recreational interest), and inattentiveness (35). Similarly, negative symptoms of ASD largely represent deficits in social–emotional reciprocity and engagement, such as reduced sharing of emotion or lack of social initiation, deficits in nonverbal communication (*e.g.*, reduced eye contact, limited use of gesture, limited range of facial expressions), and reduced spontaneous communication and conversation flow (34). These respective descriptions suggest an overlap between ASD and SZ related to the absence of typical social and communicative behaviors (9). Central to the RDoC framework, these negative symptoms that overlap in their clinical manifestation may well stem from similar biological mechanisms. In one study, participants with either ASD or SZ showed reduced neural activation in the right amygdala, fusiform face area, and ventrolateral prefrontal cortex compared to controls while performing complex social cognitive tasks (36). Thus, it is likely that negative social and communicative symptoms in both ASD and SZ have common neural origins that impact social cognition (37).

In contrast, positive symptoms in ASD and SZ may be more disorder-specific. In SZ, positive symptoms largely encompass hallucinations (*e.g.*, hearing voices that no one else hears), delusions (*e.g.*, feelings of grandiosity, or feelings of being controlled by outside forces), bizarre behavior (*e.g.*, talking to oneself, unusual dress and physical appearance), and positive formal thought disorder (*e.g.*, disorganized thinking or incoherent speech) (31, 35). Positive symptoms in ASD encompass symptomology related to speech abnormalities such as echolalia or unusual intonation, atypical social behaviors such as exaggerated gesture and facial expressions, inappropriate social overtures, and symptoms related to stereotyped and repetitive behaviors or insistence on sameness such as unusual sensory sensitivities, repetitive hand or body movements, rigid insistence on routinized behavior, or circumscribed interests (34). These respective positive symptoms in ASD and SZ overlap less than negative symptoms of each disorder do, suggesting an area of more phenotypic distinction and perhaps greater divergence in underlying neural substrates.

For the present study, we recruited community samples of adults with ASD, SZ, and typical development (TD) and administered gold-standard diagnostic interviews for both ASD and SZ. We first examined the sensitivity and specificity of the Autism Diagnostic Observation Schedule-2^nd^ edition (ADOS-2) (38) for discriminating ASD and SZ. Next, we tested whether the positive/negative framework for categorizing ASD symptomology put forth by Foss-Feig, McPartland (34) could shed light on shared and distinct clinical characteristics in ASD and SZ and improve diagnostic discrimination. We hypothesized that the ADOS-2 would have good sensitivity in ASD but poor specificity in SZ. We further expected that negative symptoms, quantified on both the ADOS-2 and the Structured Clinical Interview -Positive and Negative Syndrome Scale (SCI-PANSS) (35) would be relatively similar between ASD and SZ groups, whereas positive symptoms from each assessment would be more sensitive and disorder-specific.

## Methods

### Participants

One-hundred and thirty-two individuals participated in this study. Participants included community samples of adults with SZ (n = 39) or ASD (n = 53), aged 18–48, who were recruited for this study after seeking treatment, services, and/or research participation at the Yale Developmental Disabilities Clinic, the Specialized Treatment for Early Psychosis (STEP) Clinic, or the Yale Psychiatric department in New Haven, Connecticut. TD controls (n = 40) were recruited from the local community and from research databases. Diagnoses were confirmed by clinicians with extensive experience with both ASD and schizophrenia patients. Clinician judgments about diagnoses were informed by a variety of information, including clinician interactions with participants during administration of diagnostic assessments and prior psychiatric and medical histories obtained during recruitment procedures. Participants were excluded if they met the DSM-5 criteria for both ASD and SZ diagnosis (n = 2) as this would preclude inclusion in either group for sensitivity/specificity analyses. Participants were also excluded from analysis if they had full-scale intelligence quotient (IQ) score of less than 70 on the Wechsler Abbreviated Scale of Intelligence 2^nd^ edition (WASI-II) (39). This decision was made to ensure ability to accurately self-report during diagnostic assessments. TD participants were excluded if they had any history of a psychiatric diagnosis or if they had immediate family members with an ASD or SZ diagnosis. This study was approved by the Yale University School of Medicine Human Subject Investigation Committee. All participants provided their written informed consent to participate in this study in accordance with the Declaration of Helsinki.

Participants were matched on mean age but there were statistically significant group differences in IQ (see **Table 1**), *F*(2,129) = 13.661, *p* < .001, such that the SZ group had significantly lower IQ than the ASD group (*p* = .006) and the TD group (*p* < .001). The TD group also had higher IQ than the ASD group (*p* = .018). In addition, sex ratios between groups were unequal, χ^2^ (2, N = 132) = 10.04, *p* = .007.

**Table 1:** Sex Distributions and Means and Standard Deviations of Age and Intelligence (IQ).

	ASD (n = 53)	SZ (n = 39)	TD Controls (n = 40)
Age	24.96 (5.77)	25.77 (6.56)	26.37 (6.11)
Full-scale IQ	105.36 (16.07)	97.18 (10.38)	113.48 (13.60)
Verbal IQ	104.92 (16.78)	98.15 (11.74)	114.35 (15.90)
Nonverbal IQ	104.49 (15.89)	97.23 (10.81)	109.60 (13.08)
Sex (M, F)	(41, 12)	(32, 7)	(21, 19)

ASD, Autism Spectrum Disorder; SZ, Schizophrenia; TD, Typical Development.

### Diagnostic and Behavioral Assessments

#### ASD Symptoms

ASD symptoms were measured using the ADOS-2 (38). The ADOS-2 is a gold-standard assessment tool used to assist clinician judgment in making decisions about a possible ASD diagnosis. It is commonly used in ASD research to confirm diagnoses in a standardized manner and to estimate the severity of ASD traits (40). The ADOS-2 was administered by trained, research-reliable clinicians and consists of semistructured activities and conversations meant to sample a participant’s real-world social behavior and core autistic characteristics related to language and communication, reciprocal social interaction, imagination and creativity, stereotyped behaviors and restricted interests, and other atypical behaviors. The ADOS-2 algorithm sums across a subset of scored items in order to categorize participants into *autism* (representing higher severity ASD traits), *autism-spectrum* (representing lesser severity of ASD traits, but still enough to constitute ASD criteria), and *non-spectrum*.

#### SZ Symptoms

SZ symptoms were quantified with the Positive and Negative Syndrome Scale (PANSS) (41) after administering the SCI-PANSS interview (35). The positive symptom scale assesses the presence of delusions, conceptual disorganization, hallucinatory behavior, excitement, grandiosity, suspiciousness, and hostility. The negative symptom scale assesses blunted affect, emotional withdrawal, poor rapport, passive/apathetic social withdrawal, difficulty in abstract thinking, lack of spontaneity and conversation flow, and stereotyped thinking. Higher scores on the PANSS represent greater severity of SZ traits. Although the PANSS is intended to inform clinician judgment in considering severity of current psychosis symptoms, there are no diagnostic cut-off scores. The PANSS also has a General Psychopathology scale that measures somatic concerns, anxiety, and depression among other symptoms, but this scale was not analyzed for the purposes of the present study.

### Analysis Plan

#### Diagnostic Accuracy of ADOS

We first examined the utility of the ADOS-2 in classifying participants with ASD, SZ, or TD by comparing ADOS-2 cut-off scores with clinical diagnostic consensus by expert licensed clinical psychologists based on information obtained from the ADOS-2, SCID-R, developmental history, collateral information, and the expert opinion of licensed clinical psychologists. As reported in **Table 2**, *sensitivity* refers to the percentage of participants with ASD who met the ADOS-2 criteria for either autism or autism-spectrum. *Specificity* refers to the percentage of participants without ASD who did not meet the ADOS-2 criteria. *Positive Predictive Value* (PPV) refers to the percentage of participants who met the ADOS-2 criteria who had an ASD diagnosis. *Negative Predictive Value* (NPV) refers to the percentage of participants who did not meet the ADOS-2 criteria who also did not have an ASD diagnosis. Second, we examined Receiver Operating Characteristic (ROC) curves using ADOS scores. ROC curves offer similar sensitivity and specificity information but differ in that, instead of using algorithm cut-off scores as in **Table 2**, ROC curves examine the extent to which continuous ADOS scores correctly classify participants into DSM-5 diagnostic categories. To test our prediction that the ADOS would be more effective at discriminating the ASD and TD groups *versus* the ASD and SZ groups, we constrained the samples to just the ASD and TD groups in one analysis and just the ASD and SZ groups in a second analysis. Areas under the curve (AUC) of 1 represent perfect sensitivity and specificity of a measure, whereas.5 represents a test that is completely ineffective at discriminating diagnostic status. AUCs can be roughly interpreted as excellent = .90–1; good = .80–.90; fair = .70–.80; poor = .60–.70; bad = .50–.60 (42). All analyses on diagnostic accuracy are based on the ADOS-2 algorithm cut-off scores and the subset of items that comprise this algorithm. Sensitivity and specificity analyses were only conducted for the ADOS because there are no diagnostic cut-off scores for the PANSS.

**Table 2:** Sensitivity and Specificity of ADOS-2: Algorithm Scores.

	DSM-5 Diagnosis (clinician judgment)			
	ASD	SZ	TD	
ADOS-2 ‘autism’	27	10	0	
ADOS-2 ‘autism-spectrum’	12	7	0	PPV = 69.64%
ADOS-2 ‘non-spectrum’	14	22	40	NPV = 81.58%
	Sensitivity = 73.58%	SZ Specificity = 56.41%	TD Specificity = 100%	
		Total Specificity = 78.48%		

ADOS-2, Autism Diagnostic Observation Schedule; ASD, Autism Spectrum Disorder; SZ, Schizophrenia; TD, Typical Development; PPV, Positive Predictive Value; NPV, Negative Predictive Value.

#### Creation of Positive and Negative Symptom Domains for the ADOS

To examine our hypothesis that positive ASD symptoms would more effectively distinguish ASD and SZ, authors DT and JF grouped all ADOS-2 items (both algorithm and nonalgorithm) into positive and negative symptom categories. Examples of negative items include absence or diminished observation of typical behaviors such as absence of descriptive or instrumental gesture, deficient reporting of events, lack of communication of affect, or absence of social overtures. Positive items from the ADOS-2 include presence of atypical behaviors such as echolalia, stereotyped/idiosyncratic use of words/phrases, compulsions or rituals, or unusual sensory interests (see **Supplemental Material** for a full description of how ADOS-2 items were categorized into positive and negative symptoms). After grouping ADOS-2 items into positive and negative categories, codes indicating presence of a symptom were converted to ‘1’ and codes indicating absence of a symptom were coded as ‘0’ before being summed (see **Supplemental Material**). Total scores for ADOS-2-Positive ranged from 0 to 10, and total scores for ADOS-2-Negative ranged from 0 to 16. Six ADOS items that could not easily be categorized as positive or negative (*e.g.*, “Unusual Eye Contact,” and “Overall Quality of Rapport”) were dropped from this analysis (see *Discussion* section).

#### Comparisons of Positive and Negative Symptoms

ASD, SZ, and TD groups were then compared on positive and negative ASD symptom dimensions. We ran two separate univariate ANOVAs with diagnosis (ASD, SZ or TD) as the independent variables in both models and ASD symptom type (positive or negative) as the respective dependent variables in either model. We then examined the ROC curves of the positive and negative scales we created to examine their functioning in discriminating diagnostic groups. Finally, we completed the same steps for the PANSS, which separates symptoms into positive and negative domains by design—we conducted ANOVAs to examine diagnostic group differences in positive and negative SZ symptoms followed by analysis of ROC curves. We predicted that positive symptoms from both the ADOS and PANSS would better discriminate ASD and SZ groups than negative symptoms.

#### Controlling for IQ and Sex as Covariates

Because the SZ group had significantly lower IQ than the other groups and because groups had unequal sex distributions, we reran all main and *post hoc* analyses with PANSS and ADOS-2 (positive and negative scales) as dependent variables—this time statistically controlling for IQ and sex as covariates in ANCOVAs. These analyses were conducted to check whether the pattern of the main findings from the primary analyses was confounded by group differences in IQ and sex.

#### Intercorrelations Among PANSS and ADOS Symptom Domains

Finally, intercorrelations between negative and positive dimensions of the PANSS and ADOS were explored to examine possible content overlap among the scales.

## Results

### Diagnostic Accuracy of ADOS

#### Sensitivity and Specificity

**Table 2** displays the sensitivity, specificity, PPV, and NPV of the ADOS-2, derived by examining the proportions of participants from each subsample whose scores on the ADOS-2 accurately corresponded with the DSM-5 diagnoses determined by all the available information and clinical judgment. The specificity of the ADOS-2 was perfect in TD (100%). Of particular interest for the present study was a high rate of false positives in SZ, yielding a specificity of 56.41%. Seventeen out of 39 participants (43.59%) with a SZ diagnosis met the ADOS-2 criteria for autism or autism-spectrum despite not meeting the DSM-5 criteria for ASD by consensus diagnosis (**Table 2**).

#### Receiver Operating Characteristic Curves

**Figure 1:**
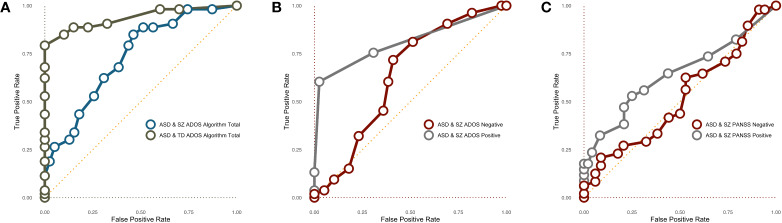
**(A)** ROC curves predicting DSM-5 diagnostic status based on continuous ADOS-2 algorithm score. **(B)** ROC curves predicting DSM-5 ASD or SZ diagnostic status based on continuous ADOS-2 negative and positive symptoms. **(C)** ROC curves predicting DSM-5 ASD or SZ diagnostic status based on continuous PANSS Negative and Positive symptoms. ASD, Autism Spectrum Disorder; SZ, Schizophrenia; TD, Typical Development.

Next, we examined several Receiver Operating Characteristic (ROC) curves using ADOS scores. The AUC for the entire sample was .84, *p* < .001, suggesting ADOS algorithm scores are a good test for discriminating ASD from the combined TD and SZ groups according to Metz’s (42) standards. In **Figure 1A**, we constrained the sample to just the ASD and TD samples or just the ASD and SZ samples. The AUC for TD + ASD was statistically significant and indicated that continuous ADOS-2 algorithm scores are an excellent tool for discriminating ASD and TD populations, AUC = .94, *p* < .001. The ROC curve for the ASD + SZ samples was also statistically significant, indicating that the ADOS-2 is able to correctly classify ASD and SZ samples; however, by Metz’s (42) standard, the AUC suggests the ADOS-2 algorithm would only be considered a “fair” test for discriminating these two populations, AUC = .73, *p* < .001.

### Comparisons of Positive and Negative Symptoms

#### ADOS

For both analyses, tests for homogeneity of variances were violated. Therefore, we ran Brown––Forsythe tests to examine equality of means and Games–Howell *post hoc* comparisons, which are more robust to homogeneity of variance violations. Diagnosis had a statistically significant effect on both positive symptoms, *F*(2,87.49) = 57.69; *p* < .001 and negative symptoms, *F*(2,110.96) = 11.83; *p* < .001. For positive symptoms, *post hoc* comparisons revealed that the ASD group displayed more positive symptoms than both the SZ (*p* < .001) and TD groups (*p* < .001), and the SZ group scored marginally higher on this scale than the TD group (*p* = .051). For negative symptoms, the ASD group scored significantly higher than the TD group (*p* < .001), and group differences between the SZ and TD groups approached significance as the SZ group scored marginally higher (*p* = .061). However, there were no statistically significant differences between the ASD and SZ groups for negative ASD symptoms (*p* = .087).

We next examined ROC curves to see if ADOS-2 positive symptoms better discriminate ASD and SZ than ADOS-2 negative symptoms. By Metz’s (42) standards, negative items poorly discriminated ASD and SZ (**Figure 1B**), AUC = .64, *p* = .03. In contrast, positive items did a good job discriminating the ASD and SZ samples, AUC = .81, *p* < .001.

#### PANSS

We next ran two univariate ANOVAs with diagnosis (ASD, SZ or TD) as the independent variable in both models and SZ symptom type (positive or negative) as the respective dependent variable. For both analyses, tests for homogeneity of variances were violated. Therefore, we ran Brown–Forsythe tests to examine quality of means and Games–Howell *post hoc* comparisons, which are more robust to homogeneity of variances violations. Diagnosis had a statistically significant effect on both positive SZ symptoms, *F*(2,50.06) = 21.75; *p* < .001 and negative SZ symptoms, *F*(2,82.92) = 45.66; *p* < .001. *Post hoc* comparisons revealed that the SZ group scored higher on positive SZ symptoms than both the ASD group (*p* = .022) and the TD group (*p*s < .001), and the ASD group scored higher than the TD group (*p* < .001). For negative SZ symptoms, the SZ group scored higher than the TD group (*p* < .001), and the ASD group also scored higher than the TD group (*p* < .001). However, there were no significant group differences between the ASD and SZ groups (*p* = .828).

**Figure 2:**
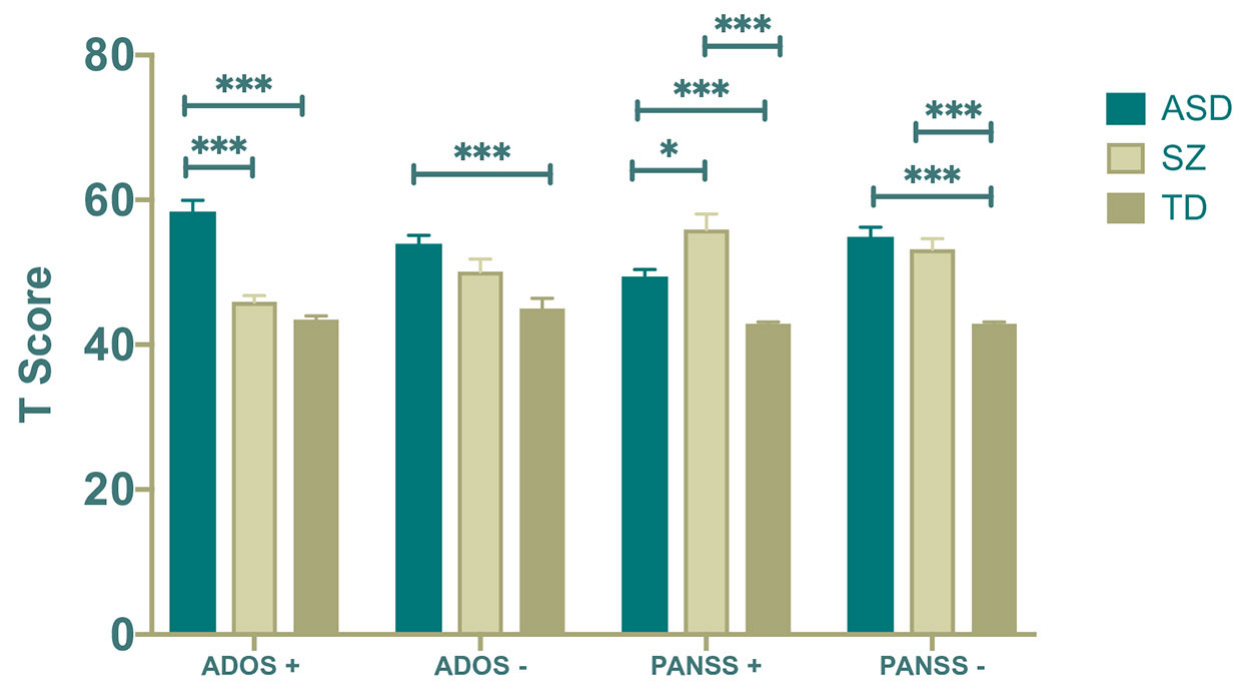
*p < .05, ***p < .001 (two-tailed). Error bars represent standard error of the means. + and − symbols refer to positive and negative symptoms, respectively. ASD, Autism Spectrum Disorder; SZ. Schizophrenia; TD, Typical Development.

We then examined ROC curves predicting diagnostic status, this time using PANSS-Negative and PANSS-Positive scores (**Figure 1C**). PANSS-Negative items measured continuously would be considered a “bad” tool for discriminating ASD and SZ groups according to Metz’s (42) standard, and the ROC curve was not statistically significant, AUC = .52, *p* = .79. In contrast, the ROC curve of PANSS-Positive was significant, AUC = .64, *p* = .030 but would still be considered a poor test in discriminating diagnostic categories. Overall, the findings suggest that positive symptoms from both the ADOS and PANSS better discriminate ASD and SZ groups than negative symptoms (see also **Figure 2**).

#### Controlling for IQ and Sex as Covariates

Because diagnosis groups had unequal IQ and sex distributions, we ran four additional ANCOVAs with diagnosis group as the independent variable, sex and IQ as covariates, and ADOS-Positive, ADOS-Negative, PANSS-Positive, and PANSS-Negative as the respective dependent variables in each ANCOVA. As the pattern of main effects and *post hoc* comparisons for all ANCOVAs after controlling for sex and IQ was the same as the original ANOVAs reported above, we do not report these analyses in detail. These analyses suggest that the key findings from our analyses were unlikely to be confounded by sex or IQ.

### Intercorrelations Among PANSS and ADOS Symptom Domains

**Table 3** shows intercorrelations between ADOS-Positive, ADOS-Negative, PANSS-Positive, and PANSS-Negative across the entire sample. Of particular interest, there was a strong, significant correlation between ADOS-Negative and PANSS-Negative, *r* = 0.58, *p* < .001, suggesting overlapping content between the two scales. In contrast, ADOS-Positive and PANSS-Positive had a low, nonsignificant correlation, *r* = 0.16, *p* = .077, suggesting the positive symptom domains from either scale are tapping relatively distinct constructs.

**Table 3:** Intercorrelations Among ADOS and PANSS Symptom Domains.

	1.	2.	3.	4.
1. ADOS-Positive		.28^**^	.16	.49^***^
2. ADOS-Negative			−.01	.58^***^
3. PANSS-Positive				.35^***^
4. PANSS-Negative				

**p < .01, ***p < .001 (two-tailed). ADOS, Autism Diagnostic Observation Scale;

PANSS, Structured Clinical Interview-Positive and Negative Syndrome Scale.

## Discussion

The key aim of this study was to examine the utility of ASD and SZ diagnostic instruments in discriminating these respective disorders. While the specificity of the ADOS-2 was perfect (100%) in classifying TD participants (true negatives), there was a high percentage of SZ false positives, such that 43.59% of participants with SZ met ADOS-2 criteria for autism or autism-spectrum despite not meeting clinical DSM-5 criteria for ASD. These findings are similar to those of Bastiaansen, Meffert (43) who found that ADOS-2 algorithm totals (44) did not significantly discriminate ASD and SZ groups in their sample, which the authors suggested was due largely to behavioral overlap between negative ASD and SZ symptoms [see also (45)].

To explore whether a subset of symptoms was driving high ADOS-2 scores in SZ participants, we categorized ADOS-2 items into positive and negative symptom domains and tested the extent to which these item clusters could better discriminate true ASD from SZ. We also examined group differences in positive and negative symptoms of SZ as measured by the PANSS. A pattern emerged such that individuals with ASD and SZ have overlap in the overt presentation of *negative* symptoms, such as reduced social–emotional reciprocity, blunted affect, reduced nonverbal communication, apathy, reduced affect sharing, and reduced social overture and response, resulting in elevated scores in both groups on the negative scales of both the ADOS-2 and PANSS. As such, though both instruments are intended to index “syndrome-specific” symptoms, due to overlapping negative symptomatology, individuals with ASD often obtain elevated scores on the PANSS and those with SZ on the ADOS-2 despite not also carrying the second diagnosis. In contrast, disorder-specific positive symptomology classified on the ADOS-2 and PANSS differentiated ASD and SZ groups more effectively. Those with SZ demonstrated higher positive symptoms related to psychosis (*e.g.*, delusions and hallucinations), whereas those with ASD demonstrated higher positive symptoms associated with ASD, including inappropriate overtures, abnormalities in language and speech, restricted interests, and repetitive behaviors. Few positive ASD symptoms were noted in SZ patients, suggesting that ratings of these symptoms may be most helpful in making a differential diagnosis in this context. This was the first time that negative and positive ASD symptoms have been split apart within a clinical measure of ASD symptoms and doing so seems to improve sensitivity and specificity.

There are two key clinical implications of this study, both related to situations where clinicians are considering a differential diagnosis between SZ and ASD. First, observing elevated score patterns across both ASD and SZ diagnostic instruments should not automatically entice clinicians to suspect dual diagnosis; instead, clinicians ought to consider whether symptoms associated with the patient’s primary disorder are leading to score inflations on measures designed for the other. Second, in order to resolve this confusion, clinicians ought to focus on the presence or absence of positive symptom domains of both ASD and SZ. Positive symptoms, especially hallucinations, delusions, grandiosity and suspiciousness may be most indicative of SZ. On the other hand, positive symptoms related to odd or excessive emotional gestures, echolalia, stereotyped speech patterns, unusual mannerisms, or circumscribed interests may be most indicative of an ASD diagnosis. Evidence of positive symptoms from both ASD and SZ diagnostic assessments may warrant a dual diagnosis, which recent research converges in suggesting occurs with more frequency than once thought (27, 46).

Findings from this study also call for increased research into the shared underlying biological systems that may give rise to ASD and SZ. Across both measures of ASD and SZ symptoms, individuals from both diagnostic categories earned elevated negative symptom scores. Previous research has identified parallel deficits in social cognition in ASD and SZ (37) that may have similar origins in atypical neural activation of select brain areas (36). There is a need for further research to understand whether the mechanisms contributing to negative symptoms in ASD and SZ are shared or distinct. Indeed, despite similar deficits in facial emotion recognition in ASD and SZ, there are markedly different patterns of EEG- and fMRI-measured neurological dysfunction associated with these deficits (46, 47). Moreover, some have questioned whether negative symptoms in schizophrenia are primary (e.g., due to true apathy and avolition) or secondary (e.g., due to depression, medication side-effects, or social avoidance due to delusional fears about social situations) (48, 49). Resolving the matter of whether negative symptoms have similar or distinct biological mechanisms is critical for determining whether ASD and SZ populations are likely to benefit from similar treatments. Likewise, better understanding the neural mechanisms of more distinct positive features of SZ and ASD may provide clues to disorder-specific pathology that could be helpful for understanding etiology, distinguishing between disorders, and developing targeted treatment.

Future research on this topic would benefit from measures that are *a priori* designed to categorize ASD symptoms into positive and negative symptoms similar to what is common practice for measuring SZ symptoms on instruments like the PANSS used in the present study. Many items on existing ASD measures do not clearly differentiate between positive and negative symptomology. In our analysis with the ADOS-2, there were 11 items where one or more of the codes could not be categorized as either positive or negative. As an example, item B1 of the ADOS-2 relates to “unusual eye contact,” which is coded when the examiner observes poorly modulated eye contact used to initiate or regulate social interactions. Eye contact could be rated “unusual” for two very different examinees: one who does not make any eye contact, and a second who stares unrelentingly. In this example, the first individual displays the absence of a typical behavior (negative symptom), whereas the second displays the presence of an atypical behavior (positive symptom). However, the rating on the ADOS-2 item as currently written is identical. Such measurement issues are not unique to the ADOS-2; in unpublished work from our group, we have found that both direct assessment and caregiver/self-report measures of ASD symptoms suffer from similar lack of specificity. As such, there is an unfortunate missed opportunity here to dissociate potentially clinically and biologically meaningful differences in behavior (see **Supplemental Material** for other ADOS-2 examples). Similar to what is already common practice in SZ research, the distinction between positive and negative symptoms may be useful for parsing heterogeneity within the ASD population, for better understanding the biology of distinct symptom manifestations and for targeting treatment. Instruments better designed to capture symptoms along these dimensions would move this goal forward.

### Limitations and Conclusions

Limitations of this study include the small sample size and unequal sex and IQ among the different diagnosis groups. While statistically controlling for sex and IQ did not change the overall pattern of results, it would have been more ideal if all groups were equal on these participant characteristics. Indeed, others have delineated the importance of carefully considering how IQ affects the results of ADOS-2 assessments (40). However, our IQ range was relatively typical of SZ samples, and lesser cognitive impairment in adults with ASD compared to SZ may more accurately represent these respective populations (50). Another limitation is that the ADOS-2 Positive and Negative items were derived *post hoc* and without a separate validation study, and many ADOS-2 items describing core ASD features could not be classified as either positive or negative so are omitted from our scales. We are not advocating for the use of the subscales we created for diagnostic purposes. Rather, our clustering provides a “proof of concept” and supports the need for new measures specifically designed and validated to distinguish positive and negative ASD symptoms. A final limitation is that, paralleling clinical activity in ASD and SZ, this study was not supported by neuroimaging, electroencephalography, or genetic data. Future research is needed to determine the biological systems that distinguish positive and negative symptom domains across ASD and SZ.

In spite of these limitations, this study has important findings adding to a body of literature demonstrating substantial symptom overlap between adults with ASD and SZ. While positive and negative SZ symptoms have long been discussed and measured in the schizophrenia literature, this study shows for the first time that distinguishing positive and negative SZ symptoms in ASD has unique value. The findings also point to the need for supplemental diagnostic measures that could more effectively parse symptom heterogeneity in ASD and better distinguish other disorders like SZ. Additional work exploring the biological overlap between ASD and SZ, as well distinguishing positive symptoms of each disorder is clearly warranted.

## Data Availability Statement

Data used in the preparation of this manuscript are publicly available to approved researchers as part of the NIMH Data Archive (nda.nih.gov) in collection C2312.

## Ethics Statement

The studies involving human participants were reviewed and approved by Yale University School of Medicine Human Subject Investigation Committee. The patients/participants provided their written informed consent to participate in this study.

## Author Contributions

DT analyzed the data and wrote the bulk of the manuscript. JF-F helped design and conceptualize the study, assessed participants, and wrote and edited sections of the manuscript. AN helped with statistical analysis and reviewed drafts of the paper. AA and VS were involved with conceptualization of the study, participant recruitment and assessment. JM oversaw all aspects of the study from study conceptualization, data collection, data analysis, and manuscript writing.

## Funding

Funding for this study was provided by NIMH R01 MH107426 (JM, VS) and NIMH R01 MH119172 (JF-F).

## Conflict of Interest

AA consults and holds equity in BlackThorn Therapeutics. JM consults with BlackThorn Therapeutics and has received research funding from Janssen Research and Development. JM also receives royalties from Guilford Press, Lambert, and Springer.

The remaining authors declare that the research was conducted in the absence of any commercial or financial relationships that could be construed as a potential conflict of interest.

The reviewer MK declared a shared affiliation, though no other collaboration, with one of the authors, JF, to the handling editor.
