# Supplementary material for: Autism Spectrum Disorder and Schizophrenia Are Better Differentiated by Positive Symptoms Than Negative Symptoms
Source: Front Psychiatry. 2020 Jun 11;11:548. doi: 10.3389/fpsyt.2020.00548 (PMC7301837; doi:10.3389/fpsyt.2020.00548)
Supplement: Supplementary file 1 [file Table_1.docx]

ADOS-2 Item Classification

| **ADOS-2 Module-4 Items** | **Positive: Presence of Atypical Behavior** | **Negative: Absence of Typical Behavior** | **Both/Neither** | **Justification** |
| --- | --- | --- | --- | --- |
| A1. Level of Non-Echoed Spoken Language |  | 1, 2, 3 |  | All codes indicate lack or absence of non-echoed complex speech |
| A2. Speech Abnormalities Associated with Autism |  |  | 1, 2, 7 | Speech abnormalities could include both negative (e.g., lack of intonation; monotone) and positive (e.g., exaggerated/unusual intonation) symptoms. |
| A3. Immediate Echolalia | 1, 2, 3 |  |  | All codes indicate presence of echoing others’ speech |
| A4. Stereotyped/Idiosyncratic use of words/phrases. | 1, 2, 3 |  |  | All codes indicate presence of stereotyped/idiosyncratic speech |
| A5. Offers Information |  | 1 | 2 | 1 code indicates ack of spontaneous offering of information. 2 code could be either negative or positive (e.g., someone who offers lots of information about a circumscribed interest). |
| A6. Asks for Information |  | 1, 2, 3 |  | Does not ask experimenter about their thoughts/feelings, etc. |
| A7. Reporting of Events |  | 1, 3 | 2 | 1 or 3 codes indicate lack of ability to report events, but 2 code could be negative, or positive (e.g., telling story that is unlikely to be true). |
| A8. Conversation |  |  | 1, 2, 3 | Lack of to-and-fro conversation flow could occur for multiple reasons. For example, an examinee could be unresponsive (negative) or continually talk over the examiner not leaving space to interject (positive). |
| A9. Descriptive, Conventional, Instrumental, or Informational Gestures. |  | 2, 3 | 1, 8 | 2 and 3 codes specify lack of gesture. 1 code addresses exaggerated (positive) OR limited (negative) use of gesture. 8 code indicates physical disability. |
| A10. Emphatic of Emotional Gestures. | 2 | 3 | 1, 8 | 1 code can indicate “exaggerated or limited” gesture (positive or negative). 2 code indicates presence of odd or excessive gesture (positive). 3 code indicates no or very limited gesture. 8 code indicates physical disability. |
| B1. Unusual Eye contact |  |  | 1, 2 | Unusual eye contact could indicate a lack of eye contact (negative symptom) or socially inappropriate eye contact (e.g., “staring”) which could be positive. |
| B2. Facial Expressions Directed to Examiner |  | 1, 2 |  | Both codes indicate lack of facial expression directed to examiner. |
| B3. Language Production and Linked Nonverbal Communication |  | 2,7 | 1, 8 | 2 code indicates lack of integrated verbal/nonverbal communication. 7 code indicates avoidance of eye gaze, 1 code indicates “abnormal” (positive) or limited (negative) integration. 8 code specifies total absence of either vocalizations or nonverbal communicative behaviors. |
| B4. Shared Enjoyment in Interaction |  | 1, 2, 3 |  | All codes indicate lack of shared enjoyment with examiner. |
| B5. Communication of Own Affect |  | 1, 2, 3 |  | All codes indicate lack of communication of affect. |
| B6. Comments on Others’ Emotions/Empathy |  | 1, 2 |  | All codes indicate lack of empathy/understanding of others’ emotions. |
| B7. Insight Into Typical Social Situations & Relationships |  |  | 1, 2, 3 | This item cannot be easily categorized into negative or positive behaviors—it is more “cognitive” in nature |
| B8. Responsibility |  |  | 1, 2 | This item is not easily categorized into negative or positive ASD behaviors. It is more indicate of adaptive functioning as opposed to a core ASD trait. |
| B9. Quality of Social Overtures. | 2 | 3 | 1 | 1 code could be lack of or unusual overture (negative or positive). 2 code indicates presence of inappropriate overtures (positive). 3 code indicates absence of social overtures (negative). |
| B10. Amount of Social Overtures/Maintenance of Attention | 7 | 1, 2, 3 |  | 1-3 codes indicate lack of overtures. 7 code indicates frequent/excessive demands for attention (positive). |
| B11. Quality of Social Response. |  | 3 | 1, 2 | 1 and 2 codes indicate lack of overtures (negative) or inappropriate/stereotyped responses (positive). 3 code specifies only lack of response to overtures. |
| B12. Amount of Reciprocal Social Communication |  | 1, 3 | 2 | 1 and 3 codes indicate lack of reciprocal social communication. The 2 code can include lack of reciprocal communication (negative) or echolalic or speech concerned with preoccupations (positive). |
| B13. Overall Quality of Rapport |  |  | 1, 2, 3 | Lack of rapport could result in for a variety of reasons, that could emerge from both positive and negative symptoms. |
| C1. Imagination/Creativity |  | 1, 3 | 2 | 1 and 3 codes indicate ack of imagination/creativity. 2 code could indicate lack of imagination/creativity (negative), but also play that’s highly repetitive or stereotyped (positive). |
| D1. Unusual Sensory Interest | 1, 2, 3 |  |  | All codes indicate presence of unusual sensory interest |
| D2. Hand and Finger/Complex mannerisms | 1, 2, 3 |  |  | All codes indicate presence of hand and finger/complex mannerisms |
| D3. Self-Injurious Behavior | 1, 2 |  |  | All codes indicate presence of self-injurious behavior |
| D4. Excessive Interest to specific topics/objects or repetitive behaviors | 1, 2, 3 |  |  | All codes indicate presence of circumscribed interests/repetitive behaviors |
| D5. Compulsions or Rituals | 1, 2 |  |  | All codes indicate presence of compulsions/rituals |
| E1. Overactivity/Agitation |  |  | 1, 2, 3, 7 | Category E represents “Other Abnormal Behaviors” that could elevate scores on the ADOS-2 but not represent core ASD characteristics. Therefore, these items were not categorized into our positive and negative ADOS-2 scales. |
| E2. Tantrums, Aggression, Negative or Disruptive Behavior |  |  | 1, 2, 3 |  |
| E3. Anxiety |  |  | 1, 2 |  |

*Note.* All codes in the Positive and Negative columns were converted to ‘1’ and summed for Positive and Negative ADOS-2 totals. Codes in the “Both/Neither” column were excluded from analyses.
